# Association of antidiabetic medication and statins with survival from ductal and lobular breast carcinoma in women with type 2 diabetes

**DOI:** 10.1038/s41598-021-88488-x

**Published:** 2021-05-17

**Authors:** Mayu Hosio, Elina Urpilainen, Ari Hautakoski, Mikko Marttila, Martti Arffman, Reijo Sund, Anne Ahtikoski, Ulla Puistola, Esa Läärä, Peeter Karihtala, Arja Jukkola

**Affiliations:** 1 Department of Oncology and Radiotherapy, Medical Research Center Oulu, Oulu University Hospital and University of Oulu, PO Box 22, 90029 Oulu, Finland; 2 Department of Obstetrics and Gynaecology, PEDEGO Research Unit, Medical Research Center Oulu, Oulu University Hospital and University of Oulu, PO Box 23, 90029 Oulu, Finland; 3 Research Unit of Mathematical Sciences, University of Oulu, PO Box 3000, 90014 Oulu, Finland; 4 Orion Corporation, Orionintie 1, PO Box 65, 02101 Espoo, Finland; 5 Service System Research Unit, Finnish Institute for Health and Welfare, PO Box 30, 00271 Helsinki, Finland; 6 Institute of Clinical Medicine, University of Eastern Finland, PO Box 1627, 70211 Kuopio, Finland; 7 Cancer and Translational Medicine Research Unit, Department of Pathology, Oulu University Hospital and University of Oulu, PO Box 50, 90029 Oulu, Finland; 8 Department of Oncology, Helsinki University Comprehensive Cancer Center, P.O.Box 180, 00029 Helsinki, Finland; 9 University of Helsinki, Helsinki, Finland; 10 Department of Oncology and Radiotherapy, Cancer Center Tampere, Tampere University Hospital, Tampere, Finland; 11 Faculty of Medicine and Health Technology, Tampere University, Box 2000, 33521 Tampere, Finland

**Keywords:** Cancer, Oncology

## Abstract

We investigated the survival of female patients with pre-existing type 2 diabetes (T2D) diagnosed with invasive ductal carcinoma (IDC) and invasive lobular carcinoma (ILC) of breast, in relation to the use of metformin, other antidiabetic medication (ADM) and statins. The study cohort consisted of 3,165 women (2,604 with IDC and 561 with ILC). The cumulative mortality from breast cancer (BC) and from other causes was calculated using the Aalen-Johansen estimator. The cause-specific mortality rates were analysed by Cox models, and adjusted hazard ratios (HRs) were estimated for the use of different medications. No evidence of an association of metformin use with BC mortality was observed in either IDC (HR 0.92, 95% confidence interval [CI] 0.64–1.31) or ILC (HR 0.68, 95% CI 0.32–1.46) patients, when compared to other oral ADMs. The mortality from other causes was found to be lower amongst the IDC patients using metformin (HR 0.64, 95% CI 0.45–0.89), but amongst ILC patients the evidence was inconclusive (HR 1.22, 95% CI 0.64–2.32). Statin use was consistently associated with reduced mortality from BC in IDC patients (HR 0.77, 95% CI 0.62–0.96) and ILC patients (HR 0.59, 95% CI 0.37–0.96), and also mortality from other causes in IDC patients (HR 0.81, 95% CI 0.67–0.96) and in ILC patients (HR 0.66, 95% CI 0.43–1.01). We found no sufficient evidence for the possible effects of metformin and statins on the prognosis of BC being different in the two histological subtypes.

## Introduction

Breast cancer (BC) is the most common cancer and an important cause of death amongst women globally^1^. Furthermore, patients with type 2 diabetes (T2D), who are diagnosed with breast cancer, have a worse prognosis^2,3^. Invasive ductal carcinoma (IDC) and invasive lobular carcinoma (ILC) are the two main histological subtypes of BC, comprising 72–80% and 5–15% of all invasive breast cancers, respectively^4,5^.

Oestrogen receptor (ER) positivity is more common in ILC, whereas human epidermal growth factor receptor 2 (HER2) overexpression is rare in this tumour type^6^. One of the main biological differences between these subtypes is the loss of expression of E-cadherin, which is observed frequently in ILC cells^7^. E-cadherin is a cell adhesion molecule expressed in normal breast tissue and is useful as a phenotypic marker in BC^8^.

ILC is more often detected in older women and is larger at diagnosis than IDC, tending to have an increased number of involved lymph nodes and indicating distinct metastatic behaviour than IDCs^9,10^. Although results on the prognosis of IDC and ILC vary, many studies have reported a similar prognosis for patients with ILC compared with IDC^10^.

Metformin is a widely prescribed oral biguanide antidiabetic medication (ADM) used as first-line therapy for patients with T2D^11^. It enhances insulin sensitivity and decreases insulin resistance^12^. Metformin use has been shown to reduce cardiovascular events in T2D patients^13,14^. The results of previous epidemiological studies on the association between metformin and survival of breast cancer patients with T2D are heterogenous^15–22^.

Statins i.e., 3-hydroxy-3-methylglutaryl-coenzyme A (HMG-CoA) reductase inhibitors, are the most prescribed medication to lower lipid level in blood. In Finland, statins are prescribed for about 80% of patients with diabetes for secondary prevention and by 40% for primary prevention of cardiovascular diseases^23^. It has been reported that statins might also have a potential anticancer role^24–27^. However, previous epidemiologic studies on statins and their association with BC prognosis are inconclusive^28–32^.

Although there are noteworthy differences in the two main histological subtypes of BC, treatment guidelines do not give specific recommendations according to the histology^33^. In this study, we investigated the survival of IDC and ILC patients in relation to the use of metformin, other ADM, and statins in women with T2D, since this question has not been addressed previously.

## Materials and methods

We have followed the Strengthening the Reporting of Observational Studies in Epidemiology (STROBE) guidelines for reporting^34^. The Diabetes in Finland database (FinDM) was used to extract the data of women with T2D. The FinDM database holds information from multiple nationwide registers, including the Special Refund Entitlement Register and the Prescription Register from the Social Insurance Institution, the Care Register for Health Care and the Hospital Discharge Register from the Finnish Institute for Health and Welfare, and the Causes of Death Register from Statistics Finland^35^.

The FinDM database includes the records of more than 240,000 women with T2D and contains reliable information on ADM and other types of medications reimbursed since 1994^35^. A patient is entered into the database at the time of the first reimbursement for ADMs or when there is a diabetes diagnosis in some of the incorporated registers, which have records dating back to 1964^35^. Data on diagnoses held in hospital records are available from 1969 onwards for inpatients and from 1998 onwards for outpatients^35^. The classification of patients into type 1 and type 2 diabetes is mainly based on the ADM used as the first-line treatment. In contrast to a local diabetes register, the FinDM data appear to have good coverage of patients with diabetes^36^. Data on cancer cases, including information on stage, were obtained by record linkage of the FinDM cohort with the Finnish Cancer Registry (FCR)^37^.

**Figure 1:**
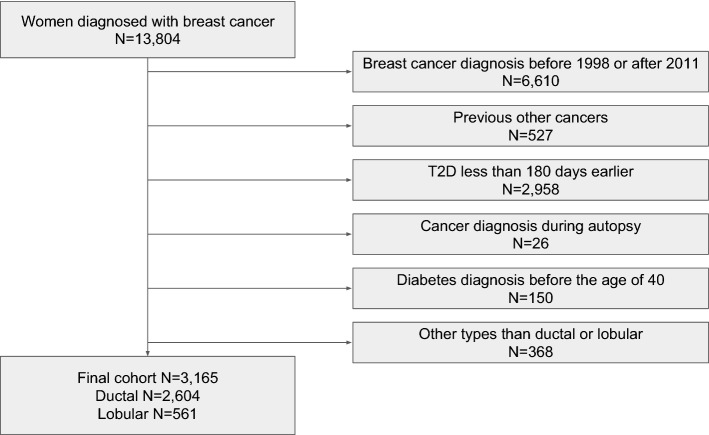
Cohort selection flowchart.

From the FinDM database, we identified 13,804 women with T2D who had also been diagnosed with BC. The additional inclusion criteria were as follows: BC diagnosed between 1998 and 2011, estimated T2D duration of at least 180 days before BC diagnosis, patients at least 40 years old when T2D was diagnosed, and the histological type of BC was ductal or lobular. Women with a prior cancer diagnosis (other than non-melanoma skin cancer) or whose BC were diagnosed at autopsy were excluded (Figure 1). Follow-up of the study cohort started at the date of diagnosis of BC and ended at the date of death, emigration or 31st of December 2013, whichever occurred first.

The patients were classified into the following mutually exclusive groups, according to their use of ADM within the three-year period before BC diagnosis: (1) metformin only; (2) other oral ADM only; (3) metformin combined with other oral ADM; (4) insulin at any time; and (5) no history of regular ADM use. Statin use was assessed in two groups: users and non-users. Exposure to all forms of medication within the three-year period before BC diagnosis was defined as starting no earlier than 180 days after the date of the first purchase. A patient who first purchased an oral ADM less than 180 days before the diagnosis of BC was categorised as having no history of regular ADM use. A single purchase of insulin within the period was enough to categorise the patient into the insulin group. A patient who had purchased a statin more than 180 days before the diagnosis of BC was categorised into the statin user group.

The FCR was used to gather the follow-up information. The dates and causes of death from the Cause of Death Register maintained by Statistics Finland are regularly linked with the FCR records. Assessment of each cancer patient’s cause of death takes into account all the data available in the FCR record, and on that basis the FCR personnel decide whether the patient died from that cancer or from some other cause. Accordingly, in this study, the causes of death were classified into two groups: death from BC and death from other causes. Information on emigration was also obtained from the FCR, as the data are linked with the Central Population Register of Finland, which holds information on individuals’ emigration and official place of residence prior to date of diagnosis^38^.

The stage of cancer at diagnosis is categorised in FCR as follows: 0) unknown, (1) localized, (2) non-localised, only regional lymph node metastases, (3) metastasised or invades adjacent tissues, (4) non-localised, no information on extent, (5) locally advanced, tumour invades adjacent tissues, and (6) non-localised, also distant lymph node metastases. Thus, the stage coding has been as follows in our study: (0) unknown, (1) local, (2–6) advanced.

The cumulative mortality from BC and from other causes was described by using the Aalen-Johansen estimator of cumulative incidence function for competing risks in the different medication groups^39,40^. To control for the effects of calendar year, age and stage at time of BC diagnosis, and T2D duration, the Cox proportional hazard models were fitted for the two causes of death separately, from which adjusted hazard ratios with 95% confidence interval were calculated. Potential differences in the HRs between the IDC and ILC patients were assessed by pertinent interaction terms in Cox models fitted for the whole cohort of BC patients. For model diagnostics, the plots of the scaled Schoenfeld residuals were visually scrutinised^41^, but no evidence for a violation of the proportional hazard’s assumption could be found that would have had any essential impact on the inference. R environment, version 3.6.1, was utilised throughout for statistical analyses^42^. The Cox models were fitted, and the assumptions were checked with the functions provided by the survival package^43^.

### Ethics declarations

All procedures performed in this study involving human participants were in accordance with the ethical standards of the Finnish national research committee and the 1964 Declaration of Helsinki and its later amendments. According to Finnish legislation, no separate ethics approval is needed for studies that involve only administrative registers. However, ethics approval was obtained for the FinDM study from the research ethics committee of the Finnish Institute of Health and Welfare (30 January 2014, meeting 1/2014, 340 §609). Permission to use data was obtained from those maintaining the original registers (the Finnish Institute for Health and Welfare, the Social Insurance Institution and Statistics Finland).

### Consent to participate

According to Finnish legislation, no separate informed consent is needed for studies that involve only administrative registers.

## Results

The final cohort included 3,165 women with BC and T2D: 2,604 with IDC and 561 with ILC (Fig. 1). The age range was 41–100 years at the time of BC diagnosis. Amongst the BC patients, 19% were classified as metformin users, 12–14% were users of other oral ADM, and 28% had no history of regular ADM use. No essential differences were found between IDC and ILC groups with regard to the use of different prediagnostic ADM (Table 1). In both the IDC and ILC groups, the patients in the insulin group had the longest duration of T2D and patients in the metformin group the shortest duration before BC diagnosis. Statins were used by 40% of the patients with IDC, and 36% with ILC. The most commonly used statins were lipophilic simvastatin and atorvastatin.

**Table 1:** Distribution of baseline characteristics and outcome status in ductal and lobular carcinoma.

	Ductal carcinoma (%)	Lobular carcinoma (%)	Total
Total n	2604	561	3165
**Age at breast cancer diagnosis (years)**			
Median (IQR^a^)	72 (64–79)	72 (64–79)	72(64–79)
40–59	392 (15)	86 (15)	478 (15)
60–69	785 (30)	169 (30)	954 (30)
70–79	825 (32)	177 (32)	1002 (32)
80–100	602 (23)	129 (23)	731 (23)
**Duration of diabetes (years)**			
Median (IQR^a^)	6.5 (3.2–10.9)	6.5 (2.9–11.3)	6.5(3.2–11.0)
0.5–3	606 (23)	142 (25)	748 (24)
3–6	612 (24)	121 (22)	733 (23)
6–12	832 (32)	183 (33)	1015 (32)
12–42	554 (21)	115 (20)	669 (21)
**Prediagnostic ADM use**			
Metformin	483 (19)	105 (19)	588 (19)
Other^b^	324 (12)	78 (14)	402 (13)
Metformin and other^b^	569 (22)	114 (20)	683 (22)
Insulin	495 (19)	105 (19)	600 (19)
No history of regular ADM^c^ use	733 (28)	159 (28)	892 (28)
**Prediagnostic statin use**			
Statin	1035 (40)	202 (36)	1237 (39)
No statin	1569 (60)	359 (64)	1928 (61)
Stage			
Local	1288 (49)	257 (46)	1545 (49)
Advanced	1143 (44)	259 (46)	1402 (44)
Unknown	173 (7)	45 (8)	218 (7)
**Outcome at the end of follow up**			
Breast cancer death	431 (17)	113 (20)	544 (17)
Other death	688 (26)	138 (25)	826 (26)
Alive	1485 (57)	310 (55)	1795 (57)

^a^Interquartile range.

^b^Other oral antidiabetic medication.

^c^ Antidiabetic medication.

**Figure 2:**
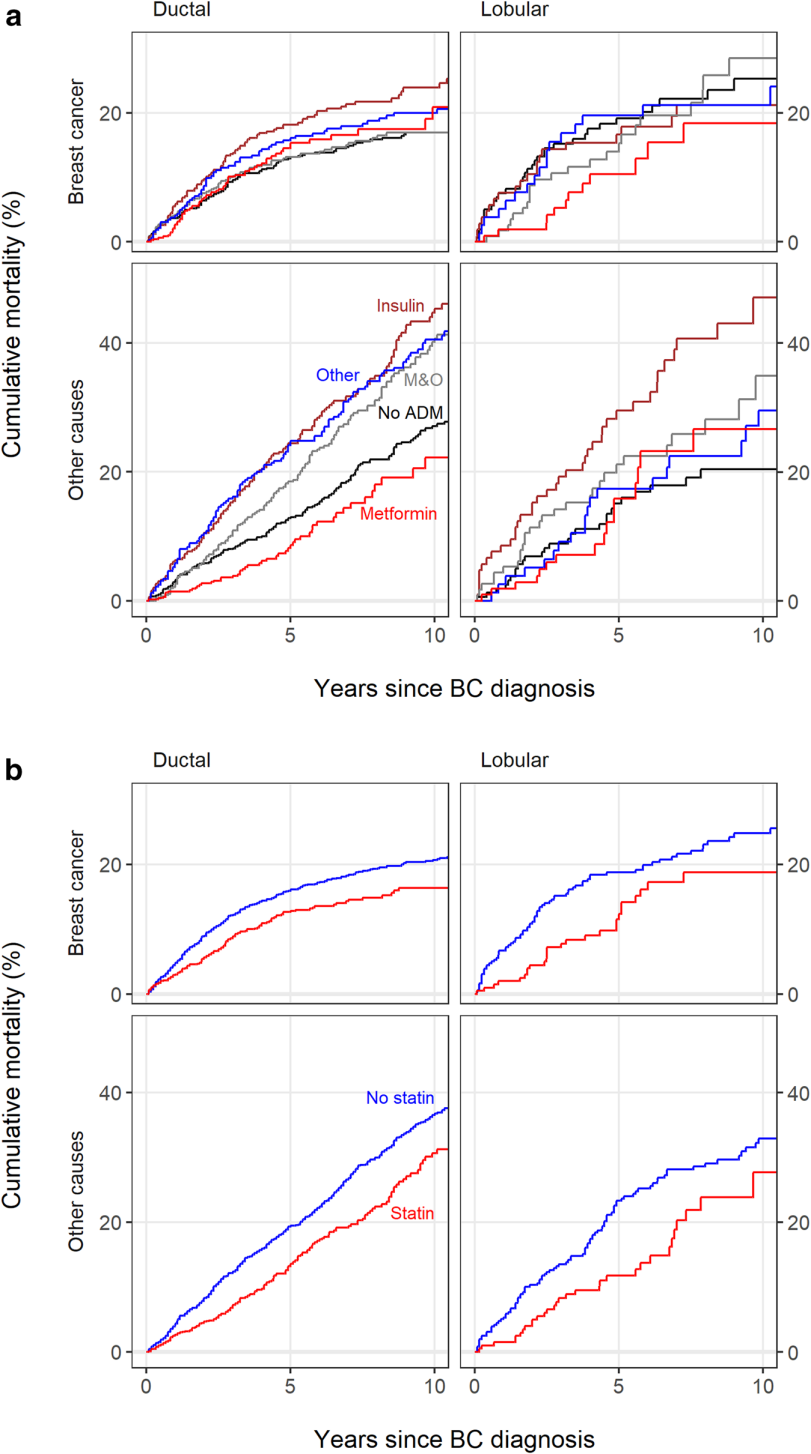
(**a**) Cumulative mortality curves in ADM groups amongst the invasive ductal and the lobular carcinoma. ADM = antidiabetic medication, M&O = metformin and other oral ADM. (**b**) Cumulative mortality curves in statin groups amongst the invasive ductal and the lobular carcinoma.

The unadjusted 10-year cumulative mortality of BC varied from 17% to 24% across the different ADM groups in the IDC patients and from 18% to 28% in the ILC patients. The unadjusted 10-year cumulative mortality of other causes varied from 22% to 45% across the different ADM groups in the IDC patients and from 20% to 47% in the ILC patients. (Figure 2a).

In the Cox proportional hazards model, a more advanced BC stage and older age were associated with increased mortality from BC in both the IDC and the ILC patients. No sufficient evidence for systematic variability in the mortality from BC was observed in the metformin users amongst either the IDC (HR 0.92, 95% CI 0.64–1.31) or the ILC (HR 0.68, 95% CI 0.32–1.46) patients. In the IDC patients, mortality from other causes of death was found to be lower amongst the metformin users (HR 0.64, 95% CI 0.45–0.89) compared to the users of other oral ADM. Amongst the ILC patients, the result was inconclusive: mortality from other causes appeared to be higher in metformin users (HR 1.22, 95% CI 0.64–2.32), but the wide confidence interval was also statistically compatible with the result in IDC patients. The mortality from other causes was higher in insulin users in both the IDC and the ILC patients compared to the users of other oral ADMs (Table 2). When analysing pertinent interactions of the studied medications with the histologic type in a joint Cox model covering all BC patients, no evidence for any HR differences between IDC and ILC could be discerned (data not shown).

**Table 2:** Estimation results from Cox proportional hazard models of mortality from the two causes of death.

	Mortality from breast cancer		Mortality from other causes	
	Ductal carcinoma	Lobular carcinoma	Ductal carcinoma	Lobular carcinoma
Variable value	Hazard ratio (95% CI)	Hazard ratio (95% CI)	Hazard ratio (95% CI)	Hazard ratio (95% CI)
**Year of diagnosis**				
1998–2002	1	1	1	1
2003–2007	0.84 (0.67–1.07)	0.98 (0.61–1.58)	0.97 (0.81–1.16)	0.98 (0.64–1.51)
2008–2011	0.96 (0.74–1.26)	1.14 (0.64–2.02)	0.80 (0.61–1.03)	0.84 (0.49–1.46)
**Age at diagnosis (years)**				
40–59	0.97 (0.69–1.37)	1.08 (0.56–2.10)	0.61 (0.41–0.92)	0.52 (0.20–1.32)
60–69	1	1	1	1
70–79	1.58 (1.22–2.04)	2.19 (1.29–3.73)	2.95 (2.33–3.75)	2.88 (1.66–5.02)
80–100	2.66 (2.01–3.50)	2.62 (1.47–4.67)	8.55 (6.70–10.9)	7.52 (4.31–13.1)
**Duration of diabetes (years)**				
0.5–3	1	1	1	1
3–6	0.89 (0.66–1.19)	0.87 (0.50–1.51)	1.05 (0.82–1.36)	0.99 (0.57–1.74)
6–12	1.00 (0.75–1.31)	0.86 (0.50–1.46)	1.34 (1.05–1.70)	0.91 (0.55–1.52)
$\ge 12$	1.08 (0.79–1.48)	0.90 (0.46–1.75)	1.23 (0.94–1.61)	1.15 (0.65–2.03)
**Stage**				
Local	1	1	1	1
Advanced	4.61 (3.64–5.83)	5.61 (3.41–9.22)	1.12 (0.95–1.32)	0.88 (0.61–1.28)
Unknown	2.07 (1.31–3.26)	2.91 (1.25–6.77)	1.51 (1.17–1.95)	0.86 (0.45–1.64)
**Prediagnostic statin use**				
No	1	1	1	1
Yes	0.77 (0.62–0.96)	0.59 (0.37–0.96)	0.81 (0.67–0.96)	0.66 (0.43–1.01)
**Prediagnostic ADM group**				
Metformin	0.92 (0.64–1.31)	0.68 (0.32–1.46)	0.64 (0.45–0.89)	1.22 (0.64–2.32)
Other^a^	1	1	1	1
Metformin and other^a^	0.76 (0.54–1.07)	1.01 (0.54–1.91)	0.93 (0.73–1.18)	1.28 (0.72–2.28)
Insulin	1.21 (0.86–1.71)	0.96 (0.47–1.95)	1.35 (1.04–1.73)	2.11 (1.14–3.91)
No history of regular ADM use	0.87 (0.63–1.19)	1.42 (0.79–2.53)	0.75 (0.59–0.95)	0.97 (0.54–1.74)

^a^ other oral antidiabetic medication.

ADM = antidiabetic medication,    95% CI    = 95% confidence interval.

Amongst the IDC patients, the 10-year cumulative BC mortality was 16% in statin users and 21% in non-users, and amongst the ILC patients these proportions were 19% and 25%, respectively. In the IDC patients, the 10-year cumulative mortality from other causes was 31% in statin users and 36% in non-users, and amongst ILC patients these proportions were 28% and 33%, respectively (Figure 2b). Prediagnostic statin use was found to be associated with decreased mortality from BC in both the IDC (HR 0.77, 95% CI 0.62–0.96) and the ILC patients (HR 0.59, 95% CI 0.37–0.96). Mortality from other causes was also observed to be decreased in both the IDC patients (HR 0.81, 95% CI 0.67–0.96) and the ILC patients (HR 0.66, 95% CI 0.43–1.01) (Table 2).

## Discussion

To the best of our knowledge, this is the first study addressing the association of ADMs or statins with survival from BC separately in the two main histological subtypes of BC. In the present study, no evidence for an association of metformin use with BC mortality was observed in either the IDC or the ILC patients when metformin use was compared with the use of other forms of oral ADM. In the IDC patients, mortality from other causes of death was found to be lower amongst metformin users compared to other oral ADM users. In the ILC patients, the result of this comparison was inconclusive due to a wide Cl, although statistically compatible with what was found for IDC patients. Insulin use was not found to have an association with BC mortality in either the IDC or the ILC patients. Prediagnostic use of statins was observed to be associated with decreased mortality from BC as well as from other causes of death in both the IDC and the ILC patients.

Insulin-like growth factor 1 (IGF-1) is a growth hormone that regulates cell growth, differentiation and transformation in various tissues, including breast tissue^44^. Increased insulin levels may induce BC carcinogenesis through crosstalk between insulin and insulin and IGF receptors, which are overexpressed in BC cells^44^. Of note, IGF-1 expression has been found to be higher in ILC than in IDC^45^, and increased IGF-1 expression has been observed to be associated with increased BC tumour size and relapse with distant metastasis, especially in ILC, but the association between IGF-expression and BC survival outcomes is variable^46–48^.

Preclinical studies have suggested that metformin could inhibit the growth of BC cells via indirect and direct pathways by reducing the level of blood glucose and insulin, which involves AMPK-dependent and -independent mechanisms^49,50^. A decrease in all-cause mortality for metformin users was reported in a meta-analysis of survival studies of BC patients with T2D, showing an overall 45% risk reduction in the total mortality rate pooled from 11 studies^51^. Vissers et al. have reported a reduced BC mortality rate only in long-term metformin users^52^.

The reference group selection is an important difference between most previous studies and ours. The reference group for metformin users was non-users of metformin in various previous studies^15–18,21,22,52,53^, while we compared metformin users to users of other oral ADMs. We assume that a reference group of ‘never used metformin’ might lead to overestimation of the possible positive association between metformin and BC survival, since this reference group also includes insulin users, whose mortality is expected to be elevated in any case^54^. There are epidemiological data that show that BC patients with T2D treated with insulin alone or sulphonylurea monotherapy had increased BC mortality^3^. Sulphonylureas are also widely used to treat T2D since they are insulin secretagogues from pancreatic $\beta$-cells^55^. This positive effect on insulin and IGF levels may progress tumorigenesis^56^. In the present study, most of the users of other oral ADM were sulphonylurea users.

Statin use reduces the risk of cardiovascular disease events in patients with T2D even without a prior history of coronary disease^57,58^. Beyond cholesterol metabolism, the mevalonate pathway is indispensable for the oncogene p53’s tumour-increasing effects^59^. Therefore, reducing mevalonate levels with statins accelerates the apoptosis of cancer cells^27,60^. Similar to our results, many studies have reported lower mortality from both BC and other causes in statin users^61–65^. However, the study populations in these studies were not limited to women with T2D. In particular, simvastatin, a highly lipophilic statin, has been associated with a decreased risk of BC recurrence or prognosis, while the association between hydrophilic statin use and BC recurrence was much more unsure^63^. Further, in a cohort study, Murtola et al. reported that simvastatin users had a decreased risk of BC mortality only amongst patients with metastatic BC at diagnosis^61^. In *in vitro* studies, only lipophilic statins were observed to inhibit BC cell proliferation^66,67^. Lipophilic statins may also penetrate the cell plasma membrane more easily than hydrophilic statins, which is possibly associated with the inhibition of cell growth^66^.

The main strength of our study is its use of nationwide database registers that hold accurate information about BC types and timing of diagnoses. The data quality in Finnish registers such as the Finnish Hospital Discharge register is regarded as high^68^. All the Nordic cancer registries have high-quality standards for the completeness and accuracy of the registered data, and patients’ causes of death are gathered from the national cause of death registries^37^. Furthermore, the Finnish Cause of Death Register’s practices and procedures comply with the coding of causes of death for mortality statistics^69^, and the FCR data enabled us to distinguish between cancer-specific and other causes of death. The duration of diabetes is regarded as precise, since it was based on the first recorded diabetes diagnosis in any of the incorporated registers or the first purchase of any form of ADM. In Finland, most forms of ADM and statins are prescribed by medical doctors and reimbursed by the Social Insurance Institution, and hence the data on the duration of medication use are accurate.

A major limitation of our study is that the data was available only from registers, which lack information on traditional prognostic factors, including hormone receptor status, lymph node status, and tumour size in different specific BC subtypes. Further, the FCR lacks accurate data on the treatment (surgery, radiation, chemotherapy, and endocrine therapy). When focusing on treatments, patients with ILC may have lower response rates to neoadjuvant chemotherapy and worse outcomes to endocrine therapies such as tamoxifen compared to patients with IDC^70,71^. The sensitivity to radiotherapy is suggested to be similar for IDC and ILC patients^72^.

Insulin is generally required in T2D treatment in the later stages of the disease, since insulin secretion decreases over time in patients with T2D^73^, and insulin treatment could represent a failure of earlier treatment or a contraindication to other types of medication, explaining general ill health^74^. Hence, different characteristics of particular medication users may induce unintentional confounding by indication in observational studies^75^, although choosing users of other ADM as the reference group, as in our study, would likely reduce this bias.

Moreover, we lacked information on lifestyle factors, such as the patients’ body mass index (BMI), smoking status, and alcohol consumption although it is known that most T2D patients are overweight or obese^76^. Some studies have reported that obesity might be independently associated with poorer prognosis for BC patients^77^, and metabolic syndrome has been found to be significantly associated with an increased risk of BC recurrence^78^. A large systematic review with meta-analysis has indicated that BC patients ceasing to smoke can lower their BC-specific mortality dramatically^79^. Previous findings regarding the association of prediagnostic alcohol consumption with breast cancer-specific mortality are mixed^80–83^. Additionally, the number of ILC patients in the final cohort was much smaller than the number of IDC patients. In consequence, the results for the ILC group were statistically less precise than those pertaining to the IDC group.

## Conclusion

No sufficient evidence could be found for metformin use being associated with a lower BC mortality compared with the use of other oral ADM in either the IDC or the ILC groups. Metformin use was observed to predict decreased mortality from other causes of death in the IDC group, but the evidence on this was inconclusive in the ILC group. Prediagnostic use of statins was found to be associated with decreased mortality from BC and from other causes in both subgroups. We found no sufficient evidence for the possible effects of metformin and statins on the prognosis of BC being different in the two major histological subtypes.

## Data Availability

The individual-level data that support the findings of this study are not publicly available for confidentiality reasons. However, aggregate data and instructions to apply for individual-level data can be requested from the FinDM database maintainers at the Finnish Institute for Health and Welfare.
